# High dose chemoradiotherapy increases chance of organ preservation with satisfactory functional outcome for rectal cancer

**DOI:** 10.1186/s13014-022-02066-7

**Published:** 2022-05-18

**Authors:** Qiao-Xuan Wang, Shu Zhang, Wei-Wei Xiao, Cheng-Jing Zhou, Hui Chang, Zhi-Fan Zeng, Pei-Qiang Cai, Zhen-Hai Lu, Gong Chen, Pei-Rong Ding, Zhi-Zhong Pan, Xiao-Jun Wu, Yuan-Hong Gao

**Affiliations:** 1 Department of Radiation Oncology, State Key Laboratory of Oncology in South China, Collaborative Innovation Center for Cancer Medicine, Sun Yat-sen University Cancer Center, 651 Dongfeng Road East, Guangzhou, 510060 People’s Republic of China; 2 Department of Medical Imaging and Interventional Radiology, State Key Laboratory of Oncology in South China, Collaborative Innovation Center for Cancer Medicine, Sun Yat-sen University Cancer Center, Guangzhou, People’s Republic of China; 3 Department of Colorectal Surgery, State Key Laboratory of Oncology in South China, Collaborative Innovation Center for Cancer Medicine, Sun Yat-sen University Cancer Center, Guangzhou, People’s Republic of China

**Keywords:** Rectal cancer, High dose chemoradiotherapy, Oncological safety, Functional outcomes, Quality of life

## Abstract

**Background:**

High dose chemoradiotherapy offers a curative chance for patients with rectal cancer that are unfit or unwilling to undergo surgical resection, yet its long-term survival and functional outcomes have been rarely investigated.

**Methods:**

Patients with non-metastatic rectal adenocarcinoma who received pelvic radiation for curative intent from April 2006 to July 2017 were retrospectively investigated. Survival rates were analyzed using the Kaplan–Meier method. Quality of life and functional outcomes were evaluated using the EORTC quality of life questionnaire.

**Results:**

A total of 57 patients were included, with a median age of 59.0 (range, 29–84) years. The numbers of patients who were diagnosed as stage I, II and III were 5 (8.8%), 16 (28.1%) and 36 (63.2%), respectively. 53 (93.0%) patients had tumor located within 5 cm from the anal verge. All patients received fluorouracil-based concurrent chemoradiotherapy with a median radiation dose of 80 (range, 60–86) Gy. All kinds of grade 3–4 adverse events occurred in 18 (31.6%) patients. 42 (73.7%) patients achieved a clinical complete response after chemoradiotherapy. After a median follow-up of 43.5 (range 14.9–163.2) months, 12 (21.1%) patients had local progression and 11 (19.3%) developed distant metastasis. The 3-year local recurrence-free survival and distant metastasis-free survival were 77.3% (95% CI, 65.7–88.8%) and 79.2% (95% CI, 68.2–90.2%), while the 3-year progression-free survival, cancer-specific survival, overall survival were 61.9% (95% CI, 48.8–75.0%), 93.1% (95% CI, 85.8–100.0%) and 91.4% (95% CI, 83.6–99.2%), respectively. For patients who had tumor located within 3 cm from the anal verge, the sphincter preservation rate was 85.3% at last follow-up. Long-term adverse events mainly were anal blood loss. 21 patients completed the quality-of-life questionnaire and had a score of the global health status of 78.57 ± 17.59. Of them, 95.2% reported no urinary incontinence and 85.7% reported no fecal incontinence.

**Conclusions:**

High dose chemoradiation demonstrated promising survival outcomes with acceptable short-term and long-term side effects, and satisfying long-term functional outcomes and quality of life. It could be considered as a non-invasive alternative for rectal cancer patients who refuse surgery.

**Supplementary Information:**

The online version contains supplementary material available at 10.1186/s13014-022-02066-7.

## Introduction

Surgery remains the essential treatment for non-metastatic rectal adenocarcinoma. However, for patients with tumor located in the distal part of the rectum, abdominoperineal resection might be necessitated in certain cases but at the expense of a permanent stoma and impaired quality of life [1]. For locally advanced rectal cancer, the combination of neoadjuvant chemoradiotherapy and surgical resection may lead to an increased risk of perioperative complications such as anastomotic leaks, postoperative anorectal, and sexual dysfunction [1–5]. For elderly patients aged over 80 years old, the 30-day postoperative mortality remains quite high, ranging between 10 and 15%, and the 6-month mortality ranges between 15 and 25% [6]. These risks of impaired functional outcomes and life-threatening postoperative complications have stimulated research on non-operative approaches for managing patients with rectal cancer.

According to previous studies [7–9], patients with rectal cancer who achieved a clinical complete response (cCR) after neoadjuvant chemoradiotherapy and managed by intensive surveillance could have similar survival outcomes, in comparison to patients who undergo standard surgery. This treatment strategy was called the watch-and-wait strategy. From the year 2004 [7] to now, the watch-and-wait strategy has been accepted by a growing number of oncologists as this has shown to be a promising therapeutic option for patients who achieved cCR. However, only approximately 15–40% of the patients could achieve cCR after neoadjuvant chemoradiotherapy [10], leaving the remaining patients with residual tumor no choice except for surgery. Therefore, increasing the probability of cCR is of great significance for patients who wish to receive the non-operative management.

Currently, most patients received long course chemoradiotherapy at the dose level of 45–50 Gy. However, a highly significant dose–response relationship was observed in rectal cancer patients who underwent neoadjuvant chemoradiotherapy [11]. This has led to growing interests for increasing the dose of radiotherapy with the goal of achieving cCR. In a study conducted by Appelt et al. [12], high dose chemoradiotherapy was found associated with an impressive cCR rate of 78.4%, indicating the possibility for further validation studies. However, the long-term survival outcome and adverse events related to high dose radiotherapy remain unclear due to limited data. Thus, we conducted this retrospective study to assess the feasibility of high dose chemoradiotherapy by analyzing the long-term survival outcome, toxicity, and quality of life of these patients.

## Materials and methods

### Patient selection

This study comprised of subjects identified from a prospective database maintained at the Sun Yat-sen University Cancer Center (Guangzhou, China) during the period of April 2006 to July 2017. All patients provided written informed consent for the collection and publication of their medical information at the first visit to our center, which was filed in their medical records. All data were retrieved from electronic data records. The inclusion criteria were as follows: (1) pathologically confirmed rectal adenocarcinoma; (2) tumor located within 10 cm from the anal verge; (3) without distant metastases; (4) receive pelvic radiation with a total dose ≥ 60 Gy for curative intent; (5) a complete set of clinical information and follow-up of more than 1 year. The study protocol was approved by the ethics committee of Sun Yat-sen University Cancer Center and was registered at ClinicalTrials.gov (identifier: NCT03541304).

### Treatment schedule

Patients in our center who received high dose chemoradiotherapy mainly underwent the following two types of processes. First, patients who refused surgery before any treatment were given one course of high dose radiotherapy, with 60–70 Gy radiation at the primary tumor site and suspected positive lymph nodes, and 45–50 Gy radiation to regional lymphatic drainage including the mesorectal, presacral, and internal iliac lymph nodes up to the bottom level of the fifth lumbar vertebra. Second, for those who had not decided whether to undergo surgery or not at the beginning of treatment, neoadjuvant chemoradiotherapy was given. The neoadjuvant radiation consisted of 45–50 Gy radiation to the primary tumor and suspected positive lymph nodes, and 45–46 Gy to regional lymphatic drainage, delivered in 25 fractions over 5 weeks. After neoadjuvant chemoradiotherapy, a second course of radiotherapy with 20–30 Gy to the primary tumor and positive lymph node was given for those patients who decided to give up surgery. Radiation was delivered in standard fraction with 6 MV photons through three-dimensional conformal radiation therapy (3D-CRT) or intensity-modulated radiation therapy (IMRT) technique. Patients lied down in a prone position, with filling of bladder. Electronic Portal Imaging Device was implied for position validation. The inductive, concurrent, and consolidative chemotherapy regimens including CapeOX (Oxaliplatin 130 mg/m^2^, day 1. Capecitabin 1000 mg/m^2^, twice daily for 14 days. Repeat every 3 weeks), mFOLFOX6 (Oxaliplatin 85 mg/m^2^, day 1. Leucovorin 400 mg/m^2^ day 1. 5-Fu 400 mg/m^2^ bolus on day 1, followed by 1200 mg/m^2^/day × 2 days. Repeat every 2 weeks), or capecitabine, prescribed at the discretion of the treating physician.

### Treatment evaluation and follow up

Pretreatment evaluation included digital rectal examination, computed tomography (CT) scan of the chest and abdomen, magnetic resonance image (MRI) of the pelvis, endorectal ultrasound or colonoscopy with a pathological examination, and serum carcinoembryonic antigen (CEA) level assessment. Treatment response was evaluated 6–8 weeks after the completion of radiation therapy and consisted of all the above-mentioned pretreatment examinations, except pathological examination. Patients were staged according to the 2010 American Joint Committee on Cancer/International Union Against Cancer (AJCC/UICC) staging system [13]. cCR after chemoradiotherapy was defined as the absence of residual tumor on digital rectal examination, pelvic MRI, and colonoscopy, accompanied by a normal CEA level, and chest and abdominal CT scan to rule out distant metastasis. Toxicities were evaluated according to the National Cancer Institute Common Toxicity Criteria 3.0 [14], and complications emerged after treatment were evaluated at post-treatment visits.

All patients were followed at 3-month intervals during the first 2 years after the completion of treatment and every 6-month thereafter for an additional period of 3 years. Digital rectal examination, CEA levels, and colonoscopy were carried out every 3 months in the first 2 years. Chest and abdominal CT scans, and pelvic MRI were performed twice a year in the first 2 years, and once every year for another 3 years. Other investigations were performed when clinically indicated during follow-up. Follow-up data, primarily obtained from the institution database, was updated by clinical chart review, physician records, patient correspondence, and telephone interviews.

### Quality of life assessment

Quality of life, toxicity and functional outcomes were evaluated for patients who were alive and without local disease progression using the European Organization for Research and Treatment of Cancer (EORTC) quality of life questionnaire, the QLQ-CR29 [15] and the QLQ-C30 modules [16]. QLQ-CR29 addressed gastrointestinal and urinary symptoms, and anorectal and sexual function. And QLQ-CR30 mainly focused on overall quality of life. The questionnaires were completed by the patients at the latest follow-up in the clinic or online on December 1st to December 31th, 2019. A standardized score was calculated according to the EORTC QLQ-CR29 and QLQ-C30 Scoring manual.

### Statistical analysis

Characteristics were described in terms of frequency for the categorical variables and medians for non-normally distributed data. Scores for the quality of life assessment were recorded as mean ± SD. The follow-up and survival periods were defined as the time span from the date of pathological diagnosis until death or censoring. Local progression was defined as a clinically proven lesion anywhere within the pelvis, either regrowth after initial decrease in size or appearance after complete remission. Distant metastasis was any tumor dissemination outside the pelvis including peritoneal carcinomatosis that occurred during follow-up. Progression-free survival (PFS) was defined as the time from diagnosis until local progression or distant metastasis, or death related to cancer. Cancer specific survival (CSS) was defined from the date of diagnosis until death from rectal cancer. Overall survival (OS) was defined from the date of diagnosis until death from any cause. Local progression-free survival (LPFS), distant metastasis-free survival (DMFS), PFS, CSS and OS were calculated using the Kaplan–Meier method and were compared using the log-rank test. Subgroup differences were examined using the log-rank test, and prognostic factors for survival were analyzed using the Cox proportional hazards regression model. All statistical tests were two-sided. Significance was set at *p* < 0.05. Statistical analyses were performed using the Statistical Package for the Social Sciences Program (SPSS Inc. Chicago, IL, version 19.0 for Windows).

## Results

### Clinical characteristics

From April 1st, 2006 to July 30, 2017, 62 patients were diagnosed with rectal cancer and received radiotherapy with a total dose ≥ 60 Gy in Sun Yat-sen University Cancer Center. Among them, five patients had metastases diseases at diagnosis and were not included in this study. Patients’ demographics and tumor characteristics were provided in Table 1. The median age of patients at diagnosis was 59.0 (range, 29–84) years. More males (77.2%) were included than female. The majority of enrolled patients were with stage II and III diseases (52, 91.2%). Nine patients had adjacent organs invasion and were defined as T4b. Most patients had their tumor located in distal rectum (46, 80.7%).

**Table 1:** Baseline clinical characteristics of patients receiving high dose chemoradiotherapy

Variable	N = 57
Age at diagnosis, n (%)	
Median, y (range)	59 (29–84)
< 60y	30 (52.6)
≥ 60y	27 (47.4)
Sex, n (%)	
Male	44 (77.2)
Female	13 (22.8)
Baseline CEA (mg/mL)	
< 5.00 mg/mL	35 (61.4)
≥ 5.00 mg/mL	22 (38.6)
T stage, n (%)	
T1	1 (1.8)
T2	10 (17.5)
T3	33 (57.9)
T4	13 (22.8)
N stage, n (%)	
N0	21 (36.8)
N1	23 (40.4)
N2	13 (22.8)
AJCC/UICC stage, n (%)	
Stage I	5 (8.8)
Stage II	16 (28.1)
Stage III	36 (63.2)
Histopathology (differentiation), n (%)	
Poorly differentiated	8 (14.0)
Moderately differentiated	33 (57.9)
Undefined	16 (28.1)
Distance to the anal verge, n (%)	
Median, cm (range)	3.0 (0.0–10.0)
≤ 5.0 cm	53 (93.0)
> 5.0 cm	4 (7.0)
Length, n (%)	
Median, cm (range)	5.0 (2.0–11.0)
< 5.0 cm	28 (49.1)
≥ 5.0 cm	29 (50.9)
Adjacent organ invasion, n (%)	
Yes	9 (15.8)
No	48 (84.2)
Comorbidities, n (%)	
Yes	10 (17.5)
No	47 (82.5)

*CEA* carcinoembryonic antigen, *AJCC/UICC* American Joint Committee on Cancer/International Union Against Cancer

### Treatment and response evaluation

Treatment details were provided in Table 2. The median radiation dose of the whole group was 80 (range, 60–86) Gy. Eleven patients who refused surgery at their first visit were given one course of radiation therapy, with a median dose of 66 (range 60–70) Gy. The other 46 patients received two courses of radiation, with a median total dose of 80 (range 66–86) Gy. For patients received two courses of radiotherapy, the most frequently used radiation dose was 50 Gy in 25 fractions in the first course (42/46, 91.3%), and 30 Gy in 15 fractions as a second boost (38/46, 82.6%). The time interval between the two courses of radiotherapy was 77 (range, 35 to 168) days. Thirteen patients had an interval longer than 12 weeks. All patients received fluorouracil-based concurrent chemotherapy. Among them, 37 (64.9%) received CapeOX, 18 (31.6%) had capecitabine monotherapy, and 2 (3.5%) were treated with the mFOLFOX6 regimen. The median cycles of chemotherapy were 8 (range, 1 to 11). 50 (87.7%) patients received ≥ 4 cycles of chemotherapy. Among these 57 patients, 45 (78.9%) refused surgery because of a permanent colostomy; eight (12.9%) patients had surgical contraindication that were deemed impossible to tolerate the operations; four (6.5%) patients were assessed as unable to achieve R0 resection after the first course of radiotherapy and therefore received a radiation boost.

**Table 2:** Treatment information, acute adverse events and response to therapy

Characteristic	No. of patients (%)
Courses of radiotherapy	
One course	11 (19.3)
Two courses	46 (80.7)
Dose of radiotherapy	
Median, Gy (range)	80 (60–86)
Radiation technology	
3D-CRT	7 (12.3)
IMRT	50 (87.7)
Chemotherapy regime	
mFolfox6	2 (3.5)
CapeOX	37 (64.9)
Capecitabine	18 (31.6)
Cycles of chemotherapy	
Median (range)	8 (1–11)
Grade 3–4 complications during treatment	
Any types	18 (31.6)
Proctitis	6 (10.5)
Diarrhea	2 (3.5)
Dermatitis associated with radiation	7 (12.3)
Leukopenia	4 (7.0)
Thrombocytopenia	6 (10.5)
Response to treatment	
cCR	42 (73.7)
Non-cCR	15 (26.3)

*3D-CRT* three-dimensional conformal radiation therapy, *IMRT* intensity-modulated radiation therapy, *cCR* clinical complete response

Acute adverse toxicity was acceptable. All patients experienced increased stool frequency (57/57), which were all classified as grade 1–2. Other commonly reported any grade complications were leukopenia (17/57), thrombocytopenia (20/57), and perianal discomfort (25/57). All kinds of grade 3–4 adverse events occurred in 18 (31.6%) patients and were provided in Table 2. Of these, the most frequently reported was radiodermatitis (12.3%), followed by thrombocytopenia (10.5%), proctitis related to radiotherapy (10.5%), leukopenia (7.0%), and diarrhea (3.5%).

All patients underwent an evaluation for treatment efficacy 6–8 weeks after the completion of the first course of radiotherapy. For patients receiving two courses of radiotherapy, reassessment was done 3 months after the finish of the second course of radiotherapy. 42 (73.7%) patients were assessed as having a cCR and 15 (26.3%) were identified as non-cCR.

### Survival of the whole group

**Fig. 1:**
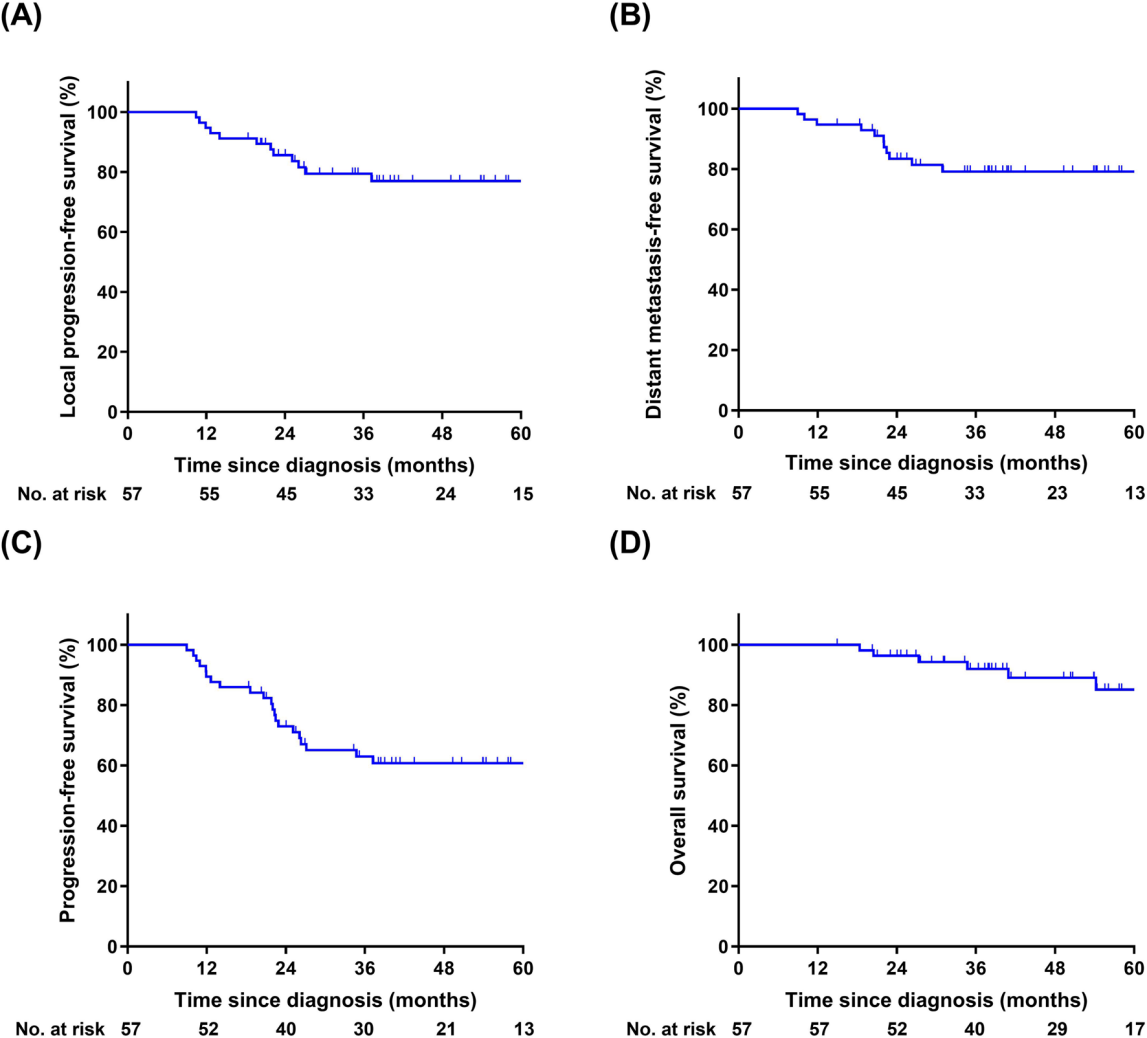
Survival of the whole cohort (N = 57). **A** Local progression-free survival; **B** distant metastasis-free survival; **C** progression-free survival; **D** overall survival

The last follow-up was on December 16, 2019, and the median follow-up time of the study was 43.5 (range 14.9–163.2) months. A total of 12 (21.1%) patients experienced local progression. Of them, three had distant metastasis detected at the same time. The median time from diagnosis to local progression was 20.7 (range, 10.4 to 37.2) months. Most local recurrences (11/12, 91.7%) occurred within the first 3 years after diagnosis. As demonstrated in Fig. 1A, 3-year and 5-year LPFS of the whole cohort were 77.3% (95% CI, 65.7–88.8%) and 77.0% (95% CI, 65.4–88.6%). Seven patients who experienced local progression underwent salvage surgery, of whom, two underwent Dixon procedure and four underwent Miles procedure. Three patients received chemotherapy, while other two patients refused further treatment and were lost to follow-up after the diagnosis of local progression. The ultimate sphincter preservation rate was 82.5% for the whole cohort. For patients who had tumor located within 3 cm from the anal verge, the sphincter preservation rate was 85.3% at last follow-up.

Eleven (19.3%) patients developed distant metastasis. Of them, eight (72.7%) patients had lung metastasis, two (18.2%) patients had liver metastasis, and one (9.1%) patient had multiple organ metastasis detected at the same time. The median time to the development of distant metastasis was 22.0 (range, 9.0–30.9) months. As shown in Fig. 1B, 3-year and 5-year DMFS of the whole cohort were 79.2% (95% CI, 68.2–90.2%), and 79.2% (95% CI, 68.2–90.2%). Five patients receive resection or ablation of metastasis with or without chemotherapy; two patients were treated by chemotherapy only; the other four patients refused further treatment and were lost to follow.

**Fig. 2:**
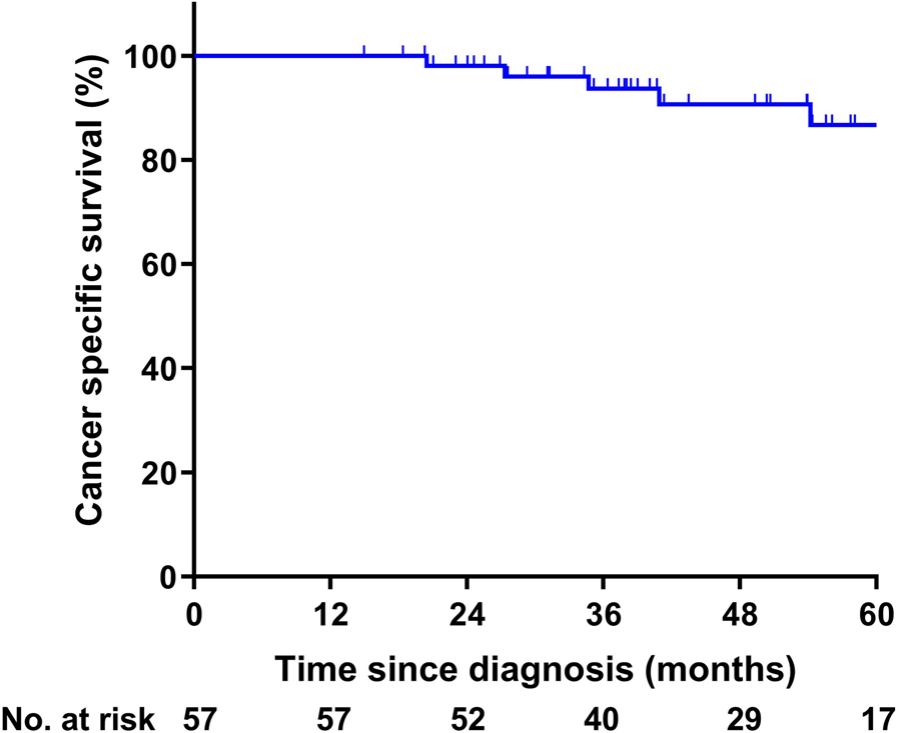
Cancer-specific survival of the whole cohort (N = 57)

The 3-year and 5-year PFS (Fig. 1C) were 61.9% (95% CI, 48.8–75.0%) and 60.8% (95% CI, 47.7–73.9%), respectively. Twelve patients died during follow-up, of whom seven died from rectal cancer and the other five died from other diseases. The 3-year and 5-year OS (Fig. 1D) of the whole cohort were 91.4% (95% CI, 83.6–99.2%) and 80.7% (95% CI, 66.4–95.0%), respectively. For there were 42.7% of deaths caused by diseases not related to rectal cancer, we calculated the CSS as another endpoint. As shown in Fig. 2, the 3-year and 5-year CSS of the whole cohort were 93.1% (95% CI, 85.8–100.0%) and 84.0% (95% CI, 70.5–97.5%), respectively.

Univariate analysis was performed to screening the factors correlating to CSS. No significant in CSS was observed regarding baseline characteristics including age, gender, TNM stage, length of tumor, histological grade, serum concentration of CEA, location of the tumor and treatment modality including courses of radiotherapy and cycles of chemotherapy (Table 3). Only response to treatment showed significant difference (*p* = 0.018, Additional file 1). Widely accepted predictors and response to treatment were selected for the multivariate analysis (Table 4), which showed that cCR after treatment had significant predictive value for CSS (cCR vs. non-cCR, HR = 16.6, *p* = 0.011). No significant difference was observed in LPFS, DMFS, PFS, OS and CSS between patients who received one and two courses of radiotherapy (Additional file 2).

**Table 3:** Univariate analysis of the risk factors for CSS (N = 57)

Variables	Univariate analysis		
	HR (95% CI)	*p* value	Log-rank *p*
Age (< 60 vs. ≥ 60)	1.308 (0.292–5.855)	0.725	0.725
Gender (male vs. female)	1.657 (0.309–8.888)	0.555	0.551
AJCC/UICC stage			0.573
I	Reference	0.603	
II	0.327 (0.02–5.240)	0.429	
III	0.966 (0.110–8.457)	0.975	
Length (< 5 vs. ≥ 5 cm)	1.308 (0.292–5.855)	0.725	0.960
Histopathology			0.629
Poorly differentiated	Reference	0.641	
Moderately differentiated	1.022 (0.105–9.920)	0.985	
Undefined	2.089 (0.216–20.179)	0.524	
CEA levels (< 5 vs. ≥ 5 ng/ml)	1.483 (0.329–6.689)	0.608	0.606
Distance to the anal verge (< 5 vs. ≥ 5 cm)	0.432 (0.051–3.645)	0.441	0.428
Courses of radiotherapy (one course vs. two course)	1.075 (0.129–8.980)	0.946	0.946
Chemotherapy regime (Capecitabine vs. Capoex/folfox)	1.005 (0.195–5.187)	0.996	0.996
Cycles of chemotherapy (≤ 4 vs. > 4)	2.052 (0.241–17.441)	0.510	0.502
Response to treatment (cCR vs. non-cCR)	6.361 (1.380–29.328)	0.018	0.007*
Comorbidities (yes vs. no)	39.757 (0.033–48,411.962)	0.310	0.083

*CSS* cancer specific survival, *HR* hazard ratio, *AJCC/UICC* American Joint Committee on Cancer/International Union Against Cancer, *CEA* carcinoembryonic antigen, *cCR* clinical complete response

*Statistically significant

**Table 4:** Multivariate analysis of the risk factors for CSS (N = 57)

Variables	Multivariate analysis	
	HR (95% CI)	*p* value
Age (< 60 vs. ≥ 60)	3.618 (0.543–24.082)	0.184
Gender (male vs. female)	4.367 (0.503–37.888)	0.181
AJCC/UICC stage		
I	Reference	0.543
II	0.176 (0.008–3.856)	0.270
III	0.384 (0.029–5.118)	0.469
Courses of radiotherapy (one course vs. two course)	0.366 (0.016–8.127)	0.525
Cycles of chemotherapy (≤ 4 vs. > 4)	3.246 (0.148–71.163)	0.455
Response to treatment (cCR vs. non-cCR)	16.616 (1.883–146.634)	0.011*

*CSS* cancer specific survival, *HR* hazard ratio, *AJCC/UICC* American Joint Committee on Cancer/International Union Against Cancer, *cCR* clinical complete response

*Statistically significant

### Long-term toxicity and quality of life

During the follow-up time, 25 (43.9%) patients had anal blood loss of any severity. Of these patients, four (7.0%) needed blood transfusion because of severe anemia. Blood loss were related to mucositis in the rectum, with most serious symptoms in the first 2 years after radiotherapy and relieved after the second year. Besides, rectum stenosis was identified in two (3.5%) cases by colonoscopy but both of them claimed no difficulty for defecation.

Patients who experienced local progression (n = 12) or death (n = 12) were exclude form quality of life assessment. Thus, 35 patients remained assessable. Of these patients, 21 returned the questionnaire, resulting in a response rate of 60%. The median follow-up time since diagnosis for these patients was 45.8 (range, 24.0–163.2) months. Of these 21 patients who completed the QLQ-C30 and the QLQ-CR29 questionnaires, 17 were male and four were female. Questions of the QLQ-C30 and QLQ-CR29 were completed for all items in 100% of the responders. Standardized scores of the questionnaires were shown in Additional file 3.

According to the QLQ-C30 questionnaires, the score of global health status/quality of life (GHS/QoL) was 78.57 ± 17.59. For functional scale questions, we defined the score above 70 as satisfactory. In general, 15 (71.4%) patients reported satisfactory overall quality of life. 85.7% of all responders reported satisfactory physical functioning and role functioning; 95.2% reported satisfactory emotional functioning; 81% reported satisfactory cognitive functioning; while 76.2% patients reported satisfactory social functioning.

**Fig. 3:**
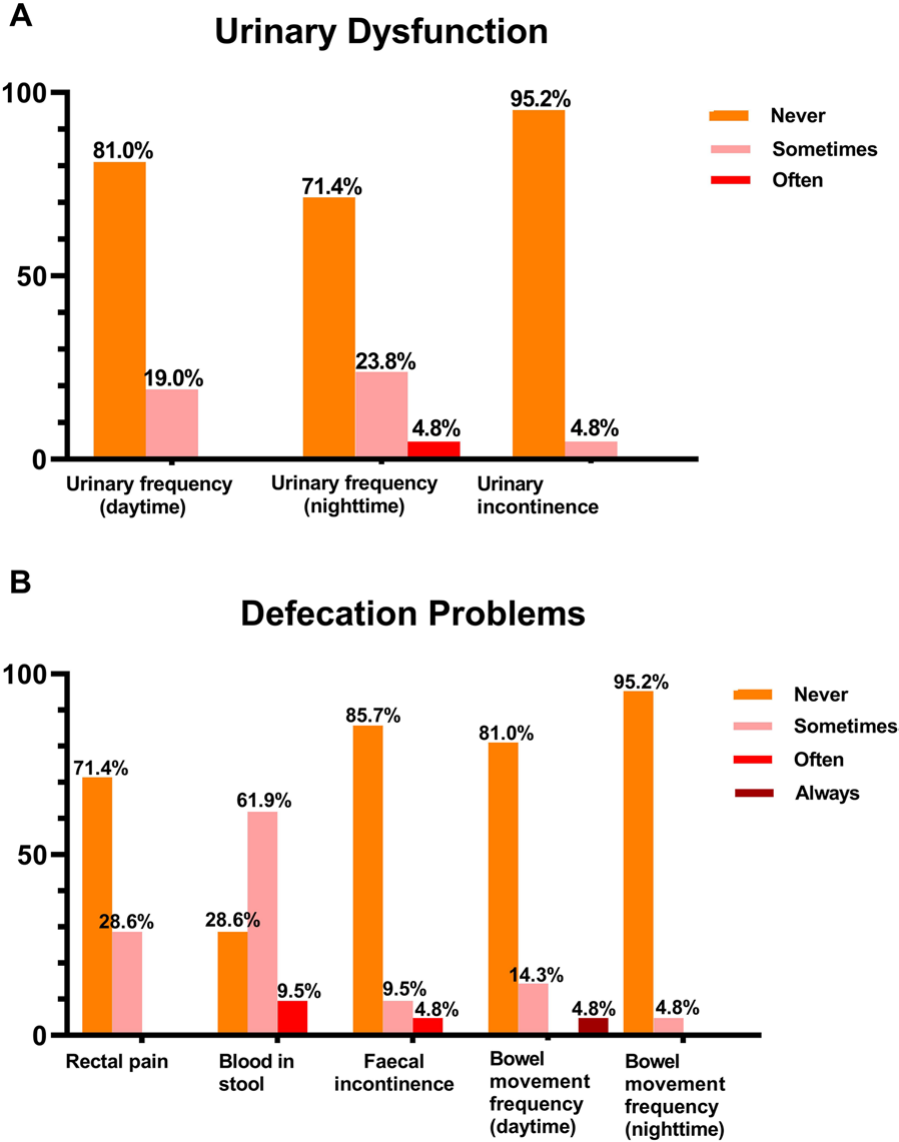
Selected patient-reported symptoms at the latest follow up according to the QLQ-CR29 questionnaires, reported as proportions of patients with symptoms of different severity. **A** Reports of urinary function related questions; **B** reports of defection related questions

For symptom and function related questions, eight (38.1%) patients reported mild symptoms of urinary frequency, while only one (4.8%) patient-reported urinary incontinences (Fig. 3A). Reports of defection related questions were shown in Fig. 3B. Fifteen (71.4%) patients reported rectal bleeding in any severity, of whom thirteen (86.7%) described it as occasional. One (4.8%) patient described the extent of symptom of fecal incontinence as moderate, while two patients (9.6%) described it as mild. The other 18 patients (85.7%) reported no such symptoms. Among 13 male patients aged < 60 years old, 8 (61.5%) preserved normal sexual function and reported no difficulty in erection; 4 (23.5%) reported a little difficulty in erection; and only one (5.9%) described it as quite a bit. Of the four female responders, one (25.0%) reported a little discomfort during intercourse, while the other three (75%) reported no such symptoms.

## Discussion

This study analyzed the clinical efficacy of patients with non-metastatic rectal cancer who underwent high dose radiotherapy with concurrent chemotherapy followed by the watch-and-wait management. An important precondition for implementing organ-sparing strategies in rectal cancer management is the complete remission of tumor after chemoradiotherapy. In order to achieve a higher cCR rate, researchers are attempting to increase the radiation doses for such patients. In line with previous studies, our study demonstrated that a high radiation dose could lead to a higher cCR rate. The cCR rate (72.6%) in this present study was comparable to previous studies. Gerard et al. [17] reported a cCR rate of 92% on high dose radiotherapy alone for T_2-3_N_0-1_M_0_ rectal cancer with 80 Gy contact X-rays, 39 Gy external beam radiotherapy and 4 Gy concomitant boost. Appelt et al. [12] showed that 78.4% of their patients with T_2-3_N_0-1_M_0_ rectal cancer achieved cCR after completion of 66 Gy of radiation and concomitant oral tegafur-uracil. These results showed that high dose radiation could provide more opportunities for patients wish to undergo the watch-and-wait strategy.

Although the local control reported in this present study was lower than previous reports with patients who underwent standard treatment [18], our findings are still satisfactory considering that all patients in our study refused to have surgery. And the 5-year local control rate of 77.0% in our study was comparable to Dizdarevic’s study on high dose chemoradiotherapy which reported a 5-year local recurrence rate of 31%. According to van der Valk’s report [19], most local regrowth occurred in the first 2 years after treatment. Thus, the follow-up time of 40 months in our study was to some extent enough to demonstrate the safety in local control of high dose radiotherapy. Meanwhile, 3-year PFS in our study (61.9%) was also comparable to Appelt’s study (2-year PFS of 58%). The overall survival in our study was slightly lower than that of patients in the studies of van der Valk et al. [19] and Dizdarevic et al. [20], which could be due to the more advanced tumor stage in some patients in current study. And there were more elderly patients in our study, who eventually died of diseases other than cancer, which could also lead to lower OS.

Apart from survival outcomes, the main concern about high dose radiotherapy was the associated adverse events. Acute adverse events were fully investigated in previous studies, mostly demonstrating a low incidence of associated adverse events [21], and was in line with our study. However, data on late toxicity were limited. In this study, we showed that the incidence of late toxicity was also relatively low. Consistent with Dizdarevic et al. [20], the most common long-term high-dose chemoradiotherapy toxicity in our study was rectal bleeding, which the researchers attributed to their administered brachytherapy boost. In our study, only an external beam boost was used, but yet rectal bleeding was still observed in some patients though most were mild. Our study also shown that mucositis resulted in rectal bleeding peaked in the first to second years after treatment, and gradually relieved after the second year. Most patients didn’t need medical intervention.

Quality of life is an important facet of cancer treatments, especially for the organ-sparing treatment strategy. Hupkens [22] demonstrated that the quality of life after successful watch-and-wait approach was better than after chemoradiation and surgery. However, there were still one-third of the watch-and-wait patients experienced major low anterior resection syndrome symptoms. In regard to high dose radiotherapy for rectal cancer, there have been few studies assessing quality of life in long-term profiles. In Dizdarevic’s study [20], high-dose chemoradiotherapy followed by nonsurgical management for distal rectal cancer showed excellent general colorectal cancer quality of life and local symptom scores. In regard to fecal incontinence, only two in 36 patients reported severe symptom (5.6%), and this was in agreement with our study (4.8%). The questionnaires collected from patients in our study also showed that most patients retained their anal, sexual, and urinary function, and most had a satisfying quality of life.

According to previous studies, approximately 15–40% of the patients could achieve cCR after neoadjuvant chemoradiotherapy [10], this meant that 50 Gy dose could be sufficient for about one-third of all rectal cancer. According to Appelt’s study [12], if we increased the dose to 65 Gy, cCR rate could be increased to around 78%. And this means that 65 Gy might be sufficient for another one-third of patients. However, it is noteworthy that some patients in our study decided to avoid surgery before treatment, while the majority made this decision after neoadjuvant chemoradiotherapy. In the former, we could make a complete plan to give one course of high dose radiation, while in the latter, there was a long interval between the two courses of radiation. It was challenging to decide the optimal radiation dose that would be effective and within the maximum dose constraints of normal tissue, as there was no suitable calculation model evaluating the biologically effective dose. In this study, the general clinical practice dealing with such problem was to prescribe 20–30 Gy boosting to the gross tumor volume, so the primary tumor would receive a total dose of 70–80 Gy. The satisfied clinical efficacy and few adverse events indicated that this dose prescribing model might be reasonable. Though there were no study giving such high dose using external beam radiation therapy in literature, there were several studies in which a second boost was given using brachytherapy after neoadjuvant chemoradiotherapy, and the total doses were as high as 75 to 80 Gy [23]. In modern era of IMRT, delivery of a high dose of radiation to the residual tumor while sparing the normal surrounding tissues is technically achievable using external beam radiation therapy. These studies together with our study, demonstrated the safety of this clinical practice.The limitations of this study were the retrospective design and small sample size. Other limitations were that the questionnaires were answered at different time points after the completion of radiotherapy for different patients and were only a one-point survey. However, according to previous report [20], overall scores of these questions at different time-points showed little variation after 2 years. As all of the patients finished the questionnaires were followed for at least 2 years, our one-point survey still preserves its power to reflect the quality of life for these patients.

## Conclusions

In conclusion, this study demonstrated that high dose chemoradiotherapy could provide high chance of organ preservation, good oncologic control with acceptable acute and chronic side effects, and satisfactory long-term quality of life. Therefore, for rectal cancer patients who refuse to undergo surgery, high dose chemoradiotherapy could be offered as a potentially curative option. Further prospective studies will be carried out to explore this treatment strategy.

## Supplementary Information

**Additional file 1.** Cancer-specific survival for patients who had clinical complete response (n = 42) after treatment and those who hadn’t (n = 15).

**Additional file 2.** Survival cures for patients who received one course of radiotherapy (n = 11) and two courses of radiotherapy (n = 46). (**A**) Local progression-free survival; (**B**) distant metastasis-free survival; (**C**) progression-free survival; (**D**) cancer specific survival; (**E**) overall survival.

**Additional file 3.** Standardized scores of the QLQ-C30 and the QLQ-CR29 questionnaires.

## Data Availability

Data of this study is available at Research Data Deposit, and the URL is https://www.researchdata.org.cn.

## Abbreviations

cCR: Clinical complete response
3D-CRT: Three-dimensional conformal radiation therapy
IMRT: Intensity-modulated radiation therapy
CT: Computed tomography
MRI: Magnetic resonance image
CEA: Carcinoembryonic antigen
AJCC: American Joint Committee on Cancer
UICC: International Union Against Cancer
EORTC: European Organization for Research and Treatment of Cancer
PFS: Progression-free survival
CSS: Cancer specific survival
OS: Overall survival
LPFS: Local progression-free survival
DMFS: Distant metastasis-free survival
