# Supplementary material for: High dose chemoradiotherapy increases chance of organ preservation with satisfactory functional outcome for rectal cancer
Source: Radiat Oncol. 2022 May 18;17:98. doi: 10.1186/s13014-022-02066-7 (PMC9118735; doi:10.1186/s13014-022-02066-7)

Additional file 1. Cancer-specific survival for patients who had clinical complete response (n=42) after treatment and those who hadn’t (n=15).


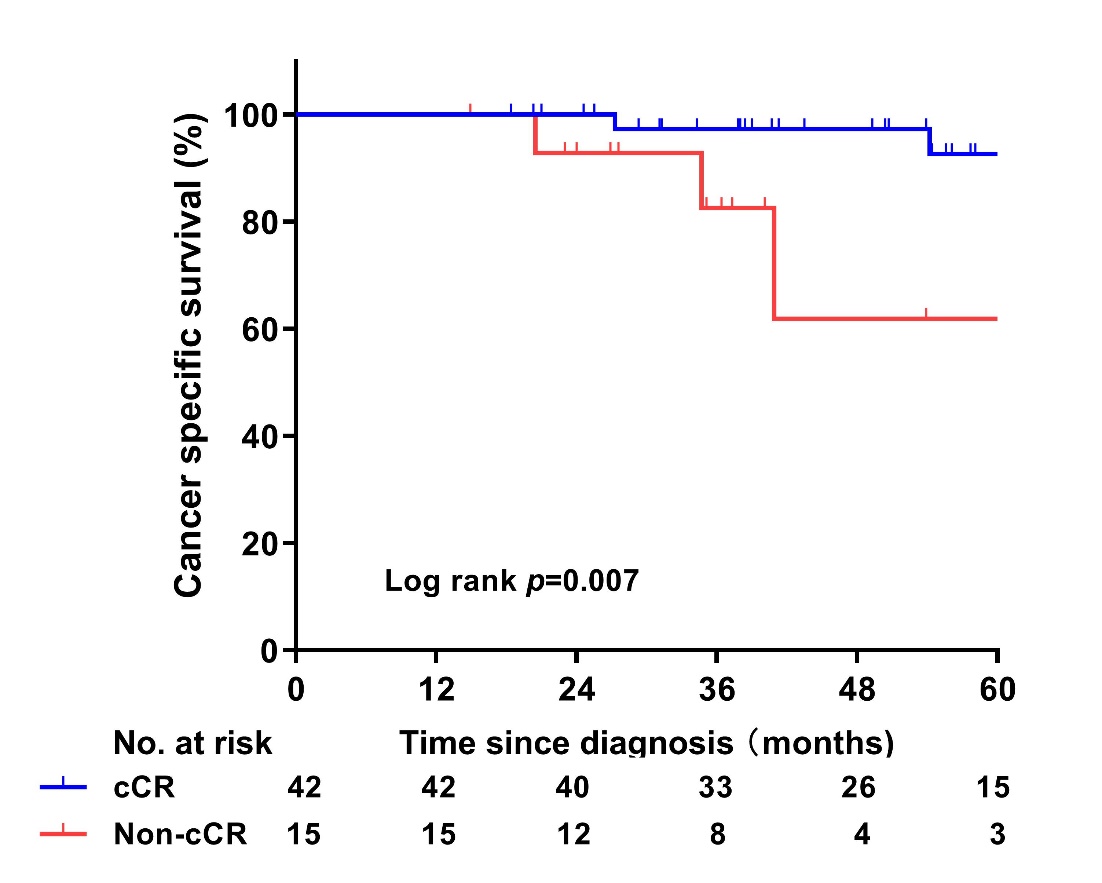

Supplement: Supplementary file 1 — Additional file 1. Cancer-specific survival for patients who had clinical complete response (n = 42) after treatment and those who hadn’t (n = 15). [file 13014_2022_2066_MOESM1_ESM.docx]
