# Supplementary material for: High dose chemoradiotherapy increases chance of organ preservation with satisfactory functional outcome for rectal cancer
Source: Radiat Oncol. 2022 May 18;17:98. doi: 10.1186/s13014-022-02066-7 (PMC9118735; doi:10.1186/s13014-022-02066-7)

Additional file 2. Survival cures for patients who received one course of radiotherapy(n=11) and two courses of radiotherapy (n=46). (A) Local progression-free survival; (B) Distant metastasis-free survival; (C) Progression-free survival; (D) Overall survival.


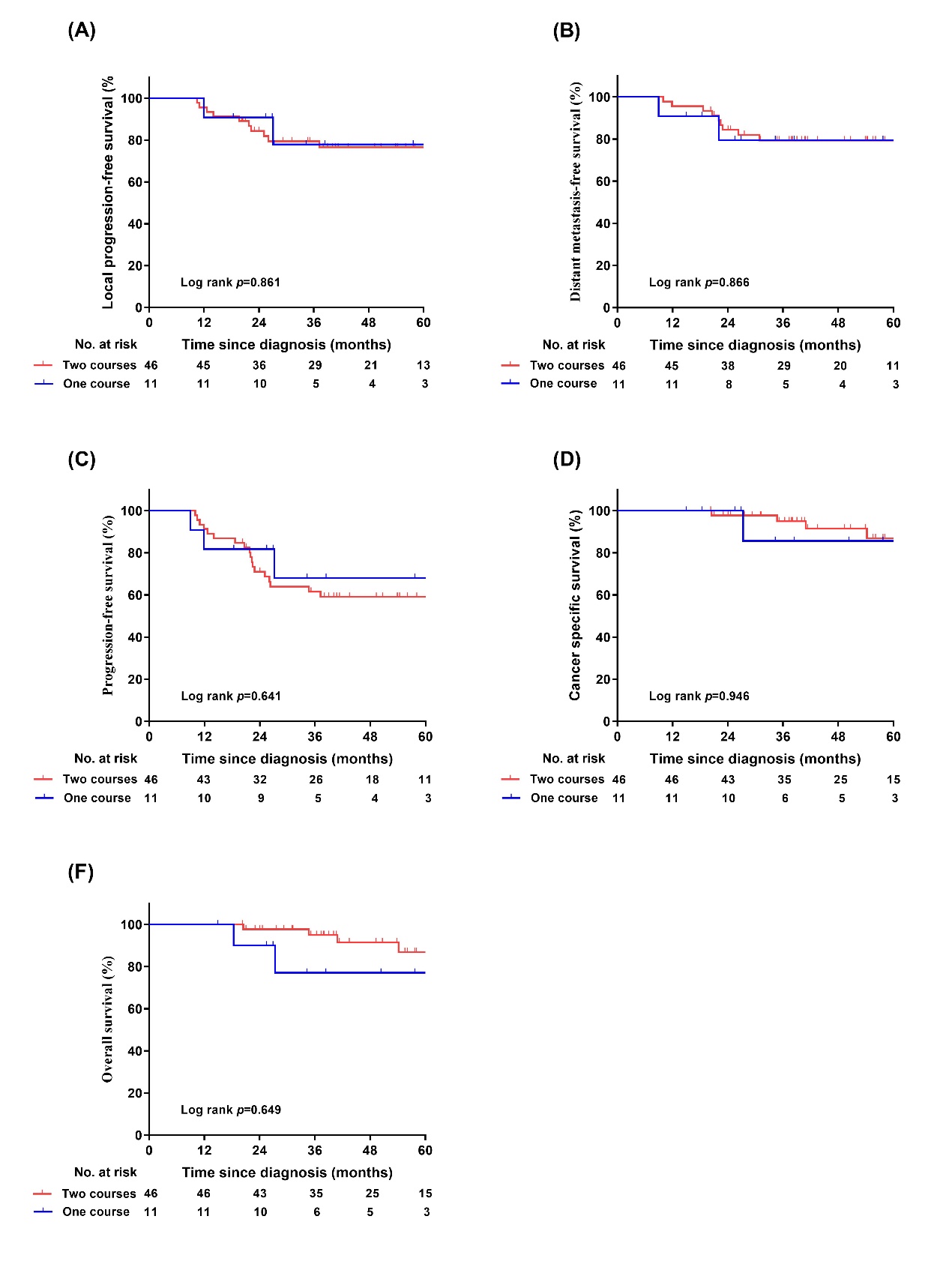

Supplement: Supplementary file 2 — Additional file 2. Survival cures for patients who received one course of radiotherapy (n = 11) and two courses of radiotherapy (n = 46). (A) Local progression-free survival; (B) distant metastasis-free survival; (C) progression-free survival; (D) cancer specific survival; (E) overall survival. [file 13014_2022_2066_MOESM2_ESM.docx]
