# Supplementary material for: High dose chemoradiotherapy increases chance of organ preservation with satisfactory functional outcome for rectal cancer
Source: Radiat Oncol. 2022 May 18;17:98. doi: 10.1186/s13014-022-02066-7 (PMC9118735; doi:10.1186/s13014-022-02066-7)
Supplement: Supplementary file 3 — Additional file 3. Standardized scores of the QLQ-C30 and the QLQ-CR29 questionnaires. [file 13014_2022_2066_MOESM3_ESM.docx]

Additional file 3. Standardized scores of the QLQ-C30 and the QLQ-CR29 questionnaires.

| ***Scales*** | n | Score (mean±SD) |
| --- | --- | --- |
| ***QLQ-C30 scales*** |  |  |
| Global health status/ QoL | 21 | 78.57±17.59 |
| Physical functioning | 21 | 92.06±13.76 |
| Role functioning | 21 | 91.27±17.17 |
| Emotional functioning | 21 | 89.68±20.23 |
| Cognitive functioning | 21 | 83.33±23.57 |
| Social functioning | 21 | 84.92±21.02 |
| Fatigue | 21 | 22.22±22.77 |
| Nausea and vomiting | 21 | 0.79±3.64 |
| Pain | 21 | 6.35±17.85 |
| Dyspnea | 21 | 11.11±19.25 |
| Insomnia | 21 | 14.29±27.02 |
| Appetite loss | 21 | 6.35±17.06 |
| Constipation | 21 | 9.52±18.69 |
| Diarrhea | 21 | 4.76±11.95 |
| Financial difficulties | 21 | 20.63±26.82 |
| ***CR29 scales*** |  |  |
| Urinary frequency | 21 | 13.49±23.93 |
| Blood and mucus in stool | 21 | 21.43±15.94 |
| Stool frequency | 21 | 5.56±15.21 |
| Body image | 21 | 15.34±19.08 |
| ***CR29 single items*** |  |  |
| Urinary incontinence | 21 | 1.59±7.27 |
| Dysuria | 21 | 0±0 |
| Abdominal pain | 21 | 3.17±10.03 |
| Buttock pain | 21 | 9.52±15.43 |
| Bloating | 21 | 3.17±10.03 |
| Dry mouth | 21 | 14.29±19.92 |
| Hair loss | 21 | 4.76±11.95 |
| Taste | 21 | 4.76±15.94 |
| Anxiety | 21 | 76.19±31.87 |
| Weight | 21 | 82.54±29.1 |
| Flatulence | 21 | 11.11±16.1 |
| Fecal incontinence | 21 | 6.35±17.06 |
| Sore skin | 21 | 11.11±19.25 |
| Embarrassment | 21 | 9.52±23.9 |
| Stoma care problems | 1 | 33.33±0 |
| Sexual interest (men) | 17 | 66.67±37.27 |
| Impotence | 17 | 17.65±29.15 |
| Sexual interest(women) | 4 | 91.67±16.67 |
| Dyspareunia | 4 | 8.33±16.67 |
